# Dietary supplements are commonly used for menopause symptoms amid concerns about hormone therapy: a cross-sectional study

**DOI:** 10.3389/fgwh.2026.1924461

**Published:** 2026-10-13

**Authors:** Susanne Mitschke

**Affiliations:** Harvard T.H. Chan School of Public Health, Boston, MA, United States

**Keywords:** dietary supplements, menopausal hormone therapy, menopause, menopause symptom management, women's health

## Abstract

**Objective:**

Many women use dietary supplements to manage menopause symptoms, yet patterns of use, perceived effectiveness, and information sources remain incompletely described in contemporary literature. This cross-sectional study assessed current dietary supplement use, including use specifically for menopause symptoms, and perceptions of menopausal hormone therapy (MHT) among symptomatic menopausal women aged 45–65 years.

**Methods:**

An online questionnaire was administered to a self-selected sample of 226 perimenopausal or postmenopausal participants recruited through online health forums, social media, menopause support groups, and newsletters. Survey items captured sociodemographic characteristics, health, supplement use, perceived effectiveness, information sources, and MHT awareness and concerns. Associations were examined using cross-tabulations, chi-square and exact tests, Spearman correlations, and logistic regression.

**Results:**

Current use of at least one dietary supplement, for any purpose, was reported by 138 participants (61.1%; 95% CI 54.6–67.2). Self-reported use of supplements specifically to manage menopause symptoms (menopause-targeted use) was reported by 65 participants (28.9%; 95% CI 23.4–35.1). Among supplement users, the most frequently reported products were multivitamins (55.8%), vitamin D (52.9%), magnesium (51.4%), and probiotics (41.3%). MHT awareness was high (*n* = 187, 82.7%) and increased with income. Concerns about breast cancer (47.8%), blood clotting (44.7%), stroke (40.7%), and heart disease (37.2%) as risks of MHT were frequently reported. Healthcare providers and online sources were the leading sources of information, and social media influencers were rated least trustworthy. Former smokers (adjusted OR 2.98, 95% CI 1.21–7.34) and never smokers (adjusted OR 3.92, 95% CI 1.59–9.65) had higher odds of any supplement use than current smokers; this association was not observed for menopause-targeted use.

**Conclusion:**

In this self-selected online sample, current dietary supplement use for any purpose was common, although fewer than half of supplement users reported using supplements to manage menopause symptoms. Concerns about MHT persisted despite high awareness. These estimates should not be generalized to the broader population of midlife women. Future studies using probability-based samples and validated symptom measures are needed to characterize menopause-targeted supplement use and to inform clinical strategies that support individualized menopause care.

## Introduction

Menopause marks a significant transition in a woman's life, typically occurring between the ages of 45 and 55, characterized by the cessation of menstrual periods for 12 consecutive months (1). This transition, known as perimenopause, is often accompanied by a variety of symptoms, including hot flashes, night sweats, mood changes, sleep disturbances, weight changes, issues with cognitive functioning, and changes in libido. These symptoms can significantly impact quality of life, prompting many women to seek effective management strategies (2).

Historically, menopausal hormone therapy (MHT), also referred to as hormone replacement therapy, has been a standard treatment for alleviating menopause symptoms (3). However, the initial results of the Women's Health Initiative (WHI), published in 2002, raised concerns about the safety of MHT, linking it to increased risks of breast cancer, heart disease, stroke, and blood clots (4). These findings led to a dramatic decline in MHT use and prompted the development of new guidelines for prescribing MHT (5). Subsequent analyses and studies have nuanced these findings, suggesting that for women younger than 60 or within 10 years of entering menopause without any contraindications, the benefits of MHT often outweigh the risks (6). Despite this, the initial WHI results have left a lasting impact on the perception and acceptance of MHT (7).

In the years following the controversy surrounding MHT, many women have turned to dietary supplements as an alternative means of managing menopause symptoms. The global menopause supplement industry is projected to reach $22.7 billion by 2028, reflecting the significant interest in non-pharmaceutical interventions (2). Popular supplements include black cohosh, vitamin E, soy isoflavones, red clover, St. John's Wort, selenium, dong quai, Chinese herbal medicine, ginseng, and evening primrose oil. Despite the variability in efficacy and safety profiles, these supplements are often perceived as natural and safe alternatives to MHT (8).

Older studies have also examined the use of dietary supplements among menopausal women. For instance, a study from 2002 found that 32% of women used dietary supplements alone, and 16% used both dietary supplements and MHT (9). Another study from 2003 showed that 46% of women used MHT and dietary supplements together, while 32% used dietary supplements alone (10). These studies indicate a significant reliance on dietary supplements among menopausal women, highlighting the need to understand current trends and practices.

This cross-sectional study aims to provide a contemporary understanding of menopause symptom management. An online questionnaire was utilized to collect data from 226 participants, exploring the prevalence of dietary supplement use among menopausal women, their perceptions of MHT, and the factors influencing their choices. The primary objective of this investigation was to estimate the prevalence of current dietary supplement use among symptomatic women aged 45–65 and the proportion who used supplements specifically to manage menopause symptoms. The secondary objectives included the exploration of perceptions on MHT, identifying sources of information about dietary supplements, and determining the factors that influence supplement choice. Demographic and socioeconomic correlates were also assessed to identify patterns and potential disparities in the use and perception of menopause treatments. By examining these areas, the present study seeks to identify current trends and influences in menopause symptom management, contributing to a broader understanding that can inform healthcare providers, policymakers, and women themselves.

## Materials and methods

### Study design and setting

This was a cross-sectional study using primary data collected between July and August 2024. The study was carried out remotely, with all screening and questionnaires completed virtually. The median time to complete the questionnaire was 2.8 min (interquartile range 2.3–4.1 min). Compensation was not provided. The study is reported in accordance with the STROBE recommendations for cross-sectional studies.

### Participants

Perimenopausal and postmenopausal participants were recruited through women's health forums and general healthcare forums, online postings on social media platforms (e.g., Facebook, Instagram), menopause support groups, and email newsletters. Perimenopause was defined as the transitional phase characterized by irregular menstrual cycles or changes in cycle length and extending until 12 months after the final menstrual period. Postmenopausal was defined as the absence of menstruation for at least 12 consecutive months. Recruitment advertisements targeted individuals interested in women's health and menopause symptom management. Study enrollment was conducted between July 21, 2024, and August 12, 2024, using voluntary response sampling to generate a nonprobability sample. Eligible participants included individuals who were aged 45–65, assigned female at birth, had access to the internet and reported experiencing menopausal or peri-menopausal symptoms.

Eligibility was self-assessed within the online questionnaire. The first item asked, “Are you currently menopausal or peri-menopausal with symptoms?”, followed by a list of example symptoms, and served as the eligibility item for menopausal status. The questionnaire did not distinguish between perimenopausal and postmenopausal status, so these groups cannot be reported separately. Age was collected in categories, and the response options included a 40–44 category that fell outside the eligibility criterion; participants who selected this category were excluded from the analytic sample.

Women with a history of breast cancer, heart disease, or blood clots were excluded because these conditions are established contraindications to MHT (6, 11). For these women, decisions about MHT are determined largely by medical restriction rather than by personal perception, information, or preference, which were the focus of this study. The exclusion therefore restricted the sample to women for whom MHT was a plausible treatment option. The consequences of this exclusion for the interpretation and generalizability of the results are discussed in the Limitations.

### Sample size

No *a priori* sample size calculation was performed. The primary aim of the study was descriptive, and recruitment was conducted over a fixed three-week period with the goal of enrolling as many eligible participants as possible. Post hoc, a sample of 226 participants provides a 95% confidence interval of approximately plus or minus 6.3 percentage points around a prevalence estimate of 61%. The analyses of associations were exploratory; the study was not powered to detect associations of a prespecified size, and the precision of subgroup estimates is reflected in the confidence intervals reported.

### Survey instrument

The questionnaire was developed for this study by the author to cover the domains of interest: sociodemographic and health characteristics, menopausal status, dietary supplement use, perceptions of MHT, and sources of information. No previously validated instrument was used. The questionnaire was reviewed by the faculty supervisor of the project and by the Harvard University Institutional Review Board as part of protocol approval. It was not pilot tested before use, which is acknowledged as a limitation. The full questionnaire is provided as Supplementary Material (Supplementary Table S5).

### Survey content

Participants completed self-report questionnaires via Qualtrics, a secure online survey platform. The questionnaire included:

a) Demographic information, such as age, race/ethnicity, and education
b) General health information, such as smoking status and family history of cancer
c) Information regarding menopausal state, such as menopause status, current menopausal or peri-menopausal symptoms
d) Prevalence of dietary supplement use, such as current use of dietary supplements for menopause symptoms, the duration and frequency of supplement use, and the types of dietary supplements used, including:
  - Vitamins and minerals (i.e., vitamin D, calcium, magnesium, multivitamins, multiminerals, or other vitamins and minerals)
  - Traditional Chinese medicine herbs
  - Herbal or botanical supplements (i.e., black cohosh, soy isoflavones, red clover, evening primrose oil, flaxseed, ginseng, St. John's Wort, valerian root, or other herbal supplements)
  - Other dietary supplements (i.e., omega-3 fatty acids, probiotics, and other dietary supplements)
e) Factors influencing supplement use, such as perceived effectiveness, safety, recommendations from healthcare providers, family and friends, social media influencers, marketing and advertisements, personal research, or other factors
f) Perceptions of MHT, such as awareness and current use of MHT and concerns about MHT, including increased risk of breast cancer, heart disease, stroke, blood clots, and other concerns

The survey was also designed to discern the primary sources of information regarding menopause symptom management, including healthcare providers, family and friends, online articles/blogs/websites, social media/influencers, books and magazines, or other sources. Trustworthiness of each source was rated on a slider labeled from 1 (not trustworthy) to 5 (very trustworthy). The slider as implemented in Qualtrics also recorded a value of 0 when it was moved to its leftmost position; values of 0 and 1 were combined and are reported as “extremely untrustworthy”, with 2 as “untrustworthy”, 3 as “neutral”, 4 as “trustworthy”, and 5 as “extremely trustworthy”.

### Definition of supplement use

Supplement use was measured with two items. Item 8 asked directly, “Are you currently using any dietary supplements to manage menopause symptoms?” (yes/no). Item 9 asked participants who use supplements to select the products they were using from a list of 20 supplement categories, with an additional option “I don't use supplements”. The two items measure different constructs and are reported as two distinct outcomes. Current supplement use (any purpose) was defined as selection of at least one supplement in item 9; because item 9 did not ask the purpose for which each product was taken, this outcome describes current supplement use regardless of the reason for use. Menopause-targeted supplement use was defined as a “yes” response to item 8. The two items were discordant for 72 participants (answered “no” to item 8 but selected at least one product, indicating current supplement use for reasons other than menopause symptoms), concordant for 65 users (“yes” and at least one product) and 88 non-users (“no” and no product), and one participant selected products but did not answer item 8. Fourteen participants left item 9 blank; all had answered “no” to item 8 and were classified as non-users of any supplement. One participant selected both a product and “I don't use supplements” and was classified as a current user. Comparisons of participant characteristics and the logistic regression were conducted with current supplement use (any purpose) as the outcome, and were repeated with menopause-targeted use as the outcome as a sensitivity analysis. Percentages for individual supplement types, duration, and frequency of use are reported with current supplement users as the denominator; percentages for perceived effectiveness and factors influencing choice are reported with current supplement users who answered the item as the denominator.

### Primary and secondary objectives

The primary objective of this cross-sectional survey was to estimate the prevalence of current dietary supplement use among symptomatic perimenopausal and postmenopausal participants and the proportion who reported using supplements specifically to manage menopause symptoms. This objective was evaluated using the two supplement-use items described above, together with items assessing the duration and frequency of supplement use and the specific types of supplements reported. The two primary outcomes were the proportion of participants reporting current use of at least one dietary supplement for any purpose and the proportion reporting menopause-targeted supplement use, overall and by supplement category.

Secondary objectives were to characterize factors associated with supplement use, describe perceptions of MHT, and evaluate participant characteristics that may influence menopause symptom management choices. Factors influencing supplement use were evaluated through survey items assessing perceived effectiveness, perceived safety, recommendations from healthcare providers, recommendations from family or friends, influence from social media, marketing or advertisements, personal research, and other reported influences. Perceptions of MHT were assessed using survey items addressing awareness of MHT, current use of MHT, and concerns about MHT, including perceived risks of breast cancer, heart disease, stroke, blood clots, and other participant-reported concerns.

Additional secondary evaluations included describing the demographic, socioeconomic, general health, and menopausal characteristics of the study population and examining their relationship with supplement use and MHT perceptions. Demographic and socioeconomic information included age, race/ethnicity, educational attainment, and annual household income. General health information included smoking status and family history of cancer. Menopausal status was assessed through the eligibility item described above. These variables were summarized descriptively and used to compare patterns of supplement use, reported reasons for use, and MHT-related perceptions across participant subgroups.

### Ethical considerations

This study involved human participants and was conducted in accordance with the principles of the Declaration of Helsinki, applicable local legislation, and institutional requirements. The study protocol, questionnaire, recruitment materials, and participant information were reviewed and approved by the Harvard University Institutional Review Board (IRB24-0917). Participation was voluntary, and participants were informed of the study purpose, procedures, potential risks, and potential benefits before beginning the online questionnaire. Participants were not required to sign a written consent form; instead, after reviewing the study information, participants indicated informed consent by selecting “I consent to participate in this study” and proceeding with the survey. Participants could withdraw at any time before submitting the questionnaire by exiting the survey. The study posed minimal risk to participants. Potential risks included discomfort from answering personal health-related questions and the possibility of breach of confidentiality. To minimize these risks, responses were collected anonymously through a secure online survey platform, and no directly identifying information was collected. Data were stored securely and were accessible only to authorized members of the research team.

### Statistical analysis

Data analysis was conducted in Python 3.12 using the pandas (3.0), SciPy (1.17), and statsmodels (0.15) libraries. Descriptive statistics were used to summarize participant characteristics and the prevalence of dietary supplement use; 95% confidence intervals for proportions were calculated with the Wilson method. Categorical variables (age group, race/ethnicity, education, income, smoking status, and family history of cancer) were compared between current supplement users (any purpose) and non-users, and between participants who were and were not aware of or using MHT, using cross-tabulations. Pearson's chi-square test (without continuity correction) was used when all expected cell counts were at least 5. When any expected cell count was below 5, Fisher's exact test was used (with the Fisher-Freeman-Halton extension for tables larger than 2 × 2, computed by complete enumeration of all tables with the observed margins). Because several race/ethnicity categories contained fewer than five participants, the association between race/ethnicity and MHT awareness was additionally examined with race/ethnicity collapsed into White/Caucasian versus all other groups.

Spearman rank correlations were used to describe the relationship between use of each individual supplement type (coded 0/1) and four ordinal participant characteristics: age group, education, household income, and smoking status (coded current smoker, former smoker, never smoked). These 80 correlations were exploratory. To account for multiple testing, false discovery rate adjusted q-values were calculated with the Benjamini-Hochberg procedure across all 80 tests, and inferential interpretation was restricted to supplement types reported by at least 10 participants. These results are presented in the Supplementary Material.

A multivariable logistic regression model was fitted with current supplement use (any purpose) as the outcome and age group, education, annual household income, smoking status, and family history of cancer as covariates, entered simultaneously. These covariates were selected *a priori* as the sociodemographic and health characteristics captured by the questionnaire that have previously been associated with complementary and alternative medicine use in menopausal women (8, 12–14). Race/ethnicity was not included because six of the seven categories contained 13 or fewer participants, which prevented stable estimation. The reference categories were 45–50 years, high school diploma/GED or less (the two participants with less than a high school education were combined with the high school diploma/GED category because a category of two participants cannot serve as a reference group), household income below $50,000, current smoker, and no family history of cancer. Adjusted odds ratios (aOR) with 95% confidence intervals and Wald *p*-values are reported, together with a likelihood ratio test for the overall model and a Wald test for the joint effect of smoking status. Multicollinearity was assessed with variance inflation factors and calibration with the Hosmer-Lemeshow test. Missing data were handled by complete-case analysis for each analysis; item-level missingness is reported in Supplementary Table S1 and the denominator is stated for each estimate. A two-sided *p*-value below 0.05 was considered statistically significant for the primary comparisons; results from the exploratory correlation analyses are interpreted in light of the adjusted q-values.

## Results

### Participant flow and characteristics

A total of 230 responses were recorded in the survey platform. One response was a test record generated through the platform's preview function before recruitment and was excluded. Three participants selected the 40–44 year age category, which fell outside the eligibility criterion, and were excluded. The analytic sample therefore comprised 226 participants (Supplementary Table S1). Of these, 215 (95.1%) answered “yes” to the menopausal status eligibility item and 11 (4.9%) left the item blank; because recruitment materials and the survey itself were explicitly directed at women experiencing menopausal or peri-menopausal symptoms, and because these 11 participants completed all remaining menopause-specific items, they were retained in the primary analysis. A sensitivity analysis excluding them (*n* = 215) produced materially unchanged results (Supplementary Table S4). One participant did not tick the consent checkbox but proceeded through the questionnaire, which under the approved consent procedure constituted consent. Item-level missingness ranged from 0% to 8.8% and was highest for the trustworthiness ratings (1.3% to 8.8%) and the supplement list item (6.2%) (Supplementary Table S1).

The majority of participants were aged between 45 and 55 (*n* = 173, 76.5%), and nearly 80% of responders identified as white or Caucasian (*n* = 180). Educational background analysis showed that 87.6% (*n* = 198) were educated with an above high school diploma or GED, and annual household income was below $100,000 in more than half of the study cohort (*n* = 141, 62.7% of 225 who reported income). Among the surveyed population, half had a history of current or past cigarette smoking (*n* = 114, 50.4%). Family history of cancer was positive in 119 (52.9% of 225) respondents (Table 1).

**Table 1:** Sociodemographic and health characteristics of the analytic sample, overall and by current supplement use for any purpose (at least one supplement type selected in item 9; *n* = 226).

Characteristic	Total *n* (%) *N* = 226	Users *n* (%) *n* = 138	Non-users *n* (%) *n* = 88	*p*-value
Age group (years)				0.055
45–50	97 (42.9%)	57 (41.3%)	40 (45.5%)	
51–55	76 (33.6%)	46 (33.3%)	30 (34.1%)	
56–60	34 (15.0%)	18 (13.0%)	16 (18.2%)	
61–65	19 (8.4%)	17 (12.3%)	2 (2.3%)	
Race or ethnicity				0.338^a^
Asian	10 (4.4%)	6 (4.3%)	4 (4.5%)	
Black/African American	13 (5.8%)	6 (4.3%)	7 (8.0%)	
Hispanic/Latino	12 (5.3%)	9 (6.5%)	3 (3.4%)	
Native American or Alaska Native	2 (0.9%)	0 (0.0%)	2 (2.3%)	
Native Hawaiian/other Pacific Islander	1 (0.4%)	0 (0.0%)	1 (1.1%)	
White/Caucasian	180 (79.6%)	112 (81.2%)	68 (77.3%)	
Other	8 (3.5%)	5 (3.6%)	3 (3.4%)	
Education				0.288^a^
Less than high school	2 (0.9%)	0 (0.0%)	2 (2.3%)	
High school diploma/GED	26 (11.5%)	17 (12.3%)	9 (10.2%)	
Some college/vocational training/Associate's degree	99 (43.8%)	56 (40.6%)	43 (48.9%)	
Bachelor's degree	56 (24.8%)	38 (27.5%)	18 (20.5%)	
Master's/Doctorate degree	43 (19.0%)	27 (19.6%)	16 (18.2%)	
Annual household income (*n* = 225)				0.712
$0 to $49,999	71 (31.6%)	40 (29.0%)	31 (35.6%)	
$50,000 to $99,999	70 (31.1%)	43 (31.2%)	27 (31.0%)	
$100,000 to $199,999	63 (28.0%)	41 (29.7%)	22 (25.3%)	
$200,000 or more	21 (9.3%)	14 (10.1%)	7 (8.0%)	
Smoking status				0.010
Current smoker	34 (15.0%)	13 (9.4%)	21 (23.9%)	
Former smoker	80 (35.4%)	50 (36.2%)	30 (34.1%)	
Never smoked	112 (49.6%)	75 (54.3%)	37 (42.0%)	
Family history of cancer (*n* = 225)				0.586
Yes	119 (52.9%)	71 (51.4%)	48 (55.2%)	
No	106 (47.1%)	67 (48.6%)	39 (44.8%)	

Percentages are column percentages. Current supplement use (any purpose) defined as selection of at least one supplement type in item 9. *p*-values from Pearson's chi-square test, or (^a^) from Fisher's exact test (Fisher-Freeman-Halton extension for tables larger than 2 × 2) where any expected cell count was below 5. Income and family history were missing for one participant each.

### Awareness, perceptions, and use of MHT

The participants' awareness and perception of MHT were evaluated within the survey. Participants were aware of MHT treatment in 82.7% (*n* = 187) cases, while 11.5% (*n* = 26) noted that they were currently using MHT for menopausal symptoms. The perception of MHT inclined towards neutral (*n* = 102, 45.1%) and somewhat positive and very positive (*n* = 83, 36.7%) responses. When asked about treatment concerns, increased risk of breast cancer (*n* = 108, 47.8%) and blood clotting (*n* = 101, 44.7%) were among the most frequently mentioned responses (Table 2). Factors associated with MHT awareness and use are shown in Table 3. Awareness was significantly associated with race/ethnicity (exact *p* = 0.001; White/Caucasian 86.1% versus all other groups 69.6%, *p* = 0.008), education (exact *p* = 0.014), annual income (exact *p* < 0.001), and smoking status (*p* = 0.010). MHT awareness rose monotonically with income, reaching 100% (21/21) among participants earning $200,000 or more, compared with 66.2% (47/71) among those earning less than $50,000. Awareness was lowest among participants identifying as Black/African American (5/13, 38.5%), participants with a high school diploma/GED (18/26, 69.2%), and current smokers (23/34, 67.6%); the single participant identifying as Native Hawaiian or other Pacific Islander was not aware of MHT, but no inference can be drawn from one observation (Table 3). Current MHT use was associated with annual income only (exact *p* = 0.003), ranging from 4.2% (3/71) among those earning less than $50,000 to 33.3% (7/21) among those earning $200,000 or more.

**Table 2:** Awareness, use, perception of, and concerns about menopausal hormone therapy (MHT) among study participants (*n* = 226).

Variable	n (%) of 226
MHT awareness	
Yes	187 (82.7%)
No	39 (17.3%)
Current use of MHT for menopausal symptoms	
Yes	26 (11.5%)
No	200 (88.5%)
Perception of MHT	
Very negative	9 (4.0%)
Somewhat negative	32 (14.2%)
Neutral	102 (45.1%)
Somewhat positive	47 (20.8%)
Very positive	36 (15.9%)
Concerns about MHT (select all that apply)	
Increased risk of breast cancer	108 (47.8%)
Increased risk of blood clots	101 (44.7%)
Increased risk of stroke	92 (40.7%)
Increased risk of heart disease	84 (37.2%)
Other concerns	15 (6.6%)
No concerns	82 (36.3%)

Concerns item: participants could select more than one option; one participant did not answer. Percentages use all 226 participants as the denominator.

**Table 3:** Participant characteristics associated with awareness and current use of menopausal hormone therapy (MHT) (*n* = 226).

Characteristic	n	Aware of MHT *n* (row %)	*p*-value	Using MHT *n* (row %)	*p*-value
Age group (years)			0.851^a^		0.696^a^
45–50	97	81 (83.5%)		13 (13.4%)	
51–55	76	64 (84.2%)		7 (9.2%)	
56–60	34	27 (79.4%)		3 (8.8%)	
61–65	19	15 (78.9%)		3 (15.8%)	
Race or ethnicity			0.001^a^		0.729^a^
Asian	10	9 (90.0%)		2 (20.0%)	
Black/African American	13	5 (38.5%)		0 (0.0%)	
Hispanic/Latino	12	10 (83.3%)		1 (8.3%)	
Native American or Alaska Native	2	2 (100.0%)		0 (0.0%)	
Native Hawaiian/other Pacific Islander	1	0 (0.0%)		0 (0.0%)	
White/Caucasian	180	155 (86.1%)		22 (12.2%)	
Other	8	6 (75.0%)		1 (12.5%)	
Education			0.014^a^		0.606^a^
Less than high school	2	2 (100.0%)		0 (0.0%)	
High school diploma/GED	26	18 (69.2%)		1 (3.8%)	
Some college/vocational training/Associate's degree	99	76 (76.8%)		11 (11.1%)	
Bachelor's degree	56	52 (92.9%)		7 (12.5%)	
Master's/Doctorate degree	43	39 (90.7%)		7 (16.3%)	
Annual household income			<0.001^a^		0.003^a^
$0 to $49,999	71	47 (66.2%)		3 (4.2%)	
$50,000 to $99,999	70	58 (82.9%)		6 (8.6%)	
$100,000 to $199,999	63	60 (95.2%)		10 (15.9%)	
$200,000 or more	21	21 (100.0%)		7 (33.3%)	
Smoking status			0.010		0.397^a^
Current smoker	34	23 (67.6%)		2 (5.9%)	
Former smoker	80	64 (80.0%)		8 (10.0%)	
Never smoked	112	100 (89.3%)		16 (14.3%)	
Family history of cancer			0.354		0.602
Yes	119	101 (84.9%)		15 (12.6%)	
No	106	85 (80.2%)		11 (10.4%)	

Row percentages: proportion within each category who were aware of MHT or currently using MHT. *p*-values from Pearson's chi-square test or (^a^) Fisher's exact test (Fisher-Freeman-Halton extension for tables larger than 2 × 2) where any expected cell count was below 5. With race/ethnicity collapsed to White/Caucasian versus all other groups, awareness was 86.1% versus 69.6% (*p* = 0.008). Income and family history were missing for one participant each. Tables 3, 4 of the original submission are combined here.

### Patterns of dietary supplement use

Current supplement use for any purpose (at least one supplement type selected in item 9) was reported by 138 of 226 participants (61.1%; 95% CI 54.6–67.2), and menopause-targeted supplement use (a “yes” response to item 8) by 65 of 225 respondents (28.9%; 95% CI 23.4–35.1) (Table 4). Thus, of the 138 current supplement users, 65 (47.1%) reported using supplements to manage menopause symptoms, 72 (52.2%) reported taking supplements but answered “no” to the menopause-specific item, and one did not answer item 8. Both estimates were essentially unchanged when the 11 participants with a blank eligibility item were excluded (60.9% and 29.4%, respectively; Supplementary Table S4).

**Table 4:** Prevalence of current dietary supplement use for any purpose, prevalence of menopause-targeted supplement use, and supplement use practices (*n* = 226).

Variable	n (%)	Denominator
Current supplement use, any purpose (item 9)		
Yes	138 (61.1%; 95% CI 54.6 to 67.2)	All participants (*n* = 226)
No	88 (38.9%)	All participants (*n* = 226)
Menopause-targeted supplement use (item 8)		
Yes	65 (28.9%; 95% CI 23.4 to 35.1)	Respondents to item 8 (*n* = 225)
No	160 (71.1%)	Respondents to item 8 (*n* = 225)
Supplement type (select all that apply)		Supplement users (*n* = 138)
Multivitamin	77 (55.8%)	[34.1% of all 226]
Vitamin D	73 (52.9%)	[32.3% of all 226]
Magnesium	71 (51.4%)	[31.4% of all 226]
Probiotics	57 (41.3%)	[25.2% of all 226]
Other vitamins	31 (22.5%)	[13.7% of all 226]
Calcium	27 (19.6%)	[11.9% of all 226]
Omega-3 fatty acids	27 (19.6%)	[11.9% of all 226]
Black cohosh	14 (10.1%)	[6.2% of all 226]
Other dietary supplement	14 (10.1%)	[6.2% of all 226]
Other herbal supplement	13 (9.4%)	[5.8% of all 226]
Multimineral	11 (8.0%)	[4.9% of all 226]
Flaxseed	9 (6.5%)	[4.0% of all 226]
Other minerals	7 (5.1%)	[3.1% of all 226]
Evening primrose oil	5 (3.6%)	[2.2% of all 226]
Traditional Chinese Medicine herbs	4 (2.9%)	[1.8% of all 226]
Red clover	4 (2.9%)	[1.8% of all 226]
Valerian root	4 (2.9%)	[1.8% of all 226]
Ginseng	3 (2.2%)	[1.3% of all 226]
Soy isoflavones	2 (1.4%)	[0.9% of all 226]
St. John's Wort	2 (1.4%)	[0.9% of all 226]
Duration of supplement use		Users who answered (*n* = 110)
Less than 1 month	6 (5.5%)	
1–3 months	13 (11.8%)	
4–6 months	15 (13.6%)	
7–12 months	17 (15.5%)	
More than 1 year	59 (53.6%)	
*Selected “I don't use supplements”*	28	Not included in denominator
Frequency of supplement use		Users who answered (*n* = 125)
Daily	111 (88.8%)	
Weekly	9 (7.2%)	
Monthly	4 (3.2%)	
Couple of times a year	1 (0.8%)	
*Selected ‘I don't use supplements’*	13	Not included in denominator

Current supplement use (any purpose): at least one supplement type selected in item 9. Menopause-targeted use: ‘yes’ to item 8 (‘Are you currently using any dietary supplements to manage menopause symptoms?’); one participant did not answer item 8. 95% CI, Wilson confidence interval. Supplement type percentages use the 138 current supplement users as the denominator; the percentage of the full analytic sample is shown in brackets for comparison with the original submission. Users could select more than one supplement type (median 3). For duration and frequency, the denominator is the number of supplement users who gave a substantive answer; users who selected ‘I don't use supplements’ on these items are shown separately. No user left these items blank.

The two groups of current users differed in the products they reported (Supplementary Table S4). Participants reporting menopause-targeted use more often reported black cohosh (18.5% versus 2.7%, *p* = 0.002), any herbal or botanical product (40.0% versus 13.7%, *p* < 0.001), and probiotics (55.4% versus 28.8%, *p* = 0.002), whereas current users who did not report menopause-targeted use more often reported multivitamins (69.9% versus 40.0%, *p* < 0.001) and calcium (26.0% versus 12.3%, *p* = 0.043).

Current supplement users (any purpose) and non-users did not differ for any tested demographic or health-related parameter, including race/ethnicity, educational level, income, and family history of cancer, except for smoking status (*p* = 0.010, Table 1); the difference across age groups did not reach significance (*p* = 0.055), although 17 of 19 participants aged 61–65 were users. No participant characteristic was associated with menopause-targeted use (item 8), including smoking status (20.6%, 27.5%, and 32.4% among current, former, and never smokers; *p* = 0.39; Supplementary Table S4).

Among the 138 current supplement users, multivitamins (*n* = 77, 55.8%), vitamin D (*n* = 73, 52.9%), magnesium (*n* = 71, 51.4%), and probiotics (*n* = 57, 41.3%) were the most commonly reported supplements, corresponding to 34.1%, 32.3%, 31.4%, and 25.2% of the full analytic sample. Soy isoflavones (*n* = 2, 1.4% of users), St. John's Wort (*n* = 2, 1.4%), ginseng (*n* = 3, 2.2%), valerian root (*n* = 4, 2.9%), traditional Chinese medicine herbs (*n* = 4, 2.9%), and red clover (*n* = 4, 2.9%) were the least frequently reported. Users reported a median of 3 supplement types. Of the 110 users who answered the duration item, 59 (53.6%) had been using supplements for more than a year; of the 125 users who answered the frequency item, 111 (88.8%) reported daily use (Table 4). Twenty-eight users (20.3%) selected “I don't use supplements” on the duration item and 13 (9.4%) on the frequency item; these participants were predominantly those who had answered “no” to the menopause-specific item 8.

### Factors influencing supplement choice, perceived effectiveness, and information sources

Table 5 summarizes factors influencing supplement choice, perceived effectiveness, and the trustworthiness of information sources. Of the 113 supplement users who answered the item, 48 (42.5%) identified personal research as the main factor influencing their choice of supplement, and 33 (29.2%) identified recommendations from healthcare providers. Participants rated the perceived effectiveness of their supplements from “very ineffective” to “very effective”. Of the 115 supplement users who answered this item, 54 (47.0%) rated their supplements as somewhat effective (*n* = 47, 40.9%) or very effective (*n* = 7, 6.1%), 32 (27.8%) were neutral, and 29 (25.2%) rated them as somewhat ineffective (*n* = 14, 12.2%) or very ineffective (*n* = 15, 13.0%). Perceived effectiveness was higher among users reporting menopause-targeted use [36 (56.2%) of 64 who answered] than among users who did not [18 (35.3%) of 51; Mann–Whitney *p* = 0.015].

**Table 5:** Factors influencing supplement choice, perceived effectiveness, information sources, and trustworthiness of information sources.

Variable	n (%)	Denominator
Main factor influencing supplement choice		Users who answered (*n* = 113)
Personal research	48 (42.5%)	
Recommendations from healthcare providers	33 (29.2%)	
Perceived efficacy	11 (9.7%)	
Recommendations from family and friends	7 (6.2%)	
Safety	5 (4.4%)	
Recommendations from social media influencers	1 (0.9%)	
Marketing and advertisements	1 (0.9%)	
Other	7 (6.2%)	
*Selected ‘I don't use supplements’*	25	Not included
Perceived effectiveness of supplements (overall)		Users who answered (*n* = 115)
Very ineffective	15 (13.0%)	
Somewhat ineffective	14 (12.2%)	
Neutral	32 (27.8%)	
Somewhat effective	47 (40.9%)	
Very effective	7 (6.1%)	
*Selected ‘I don't use supplements’*	23	Not included
Primary sources of information (select all that apply)		All participants (*n* = 226)
Healthcare providers	156 (69.0%)	
Internet, online articles, blogs, and websites	153 (67.7%)	
Family and friends	97 (42.9%)	
Books and magazines	53 (23.5%)	
Social media and influencers	45 (19.9%)	
Other	3 (1.3%)	
Trustworthiness: Healthcare providers		Participants who rated (*n* = 223)
Extremely untrustworthy	8 (3.6%)	
Untrustworthy	8 (3.6%)	
Neutral	39 (17.5%)	
Trustworthy	92 (41.3%)	
Extremely trustworthy	76 (34.1%)	
Trustworthiness: Family and friends		Participants who rated (*n* = 218)
Extremely untrustworthy	23 (10.6%)	
Untrustworthy	38 (17.4%)	
Neutral	81 (37.2%)	
Trustworthy	54 (24.8%)	
Extremely trustworthy	22 (10.1%)	
Trustworthiness: Internet, online articles, blogs, and websites		Participants who rated (*n* = 221)
Extremely untrustworthy	27 (12.2%)	
Untrustworthy	37 (16.7%)	
Neutral	90 (40.7%)	
Trustworthy	53 (24.0%)	
Extremely trustworthy	14 (6.3%)	
Trustworthiness: Social media and influencers		Participants who rated (*n* = 206)
Extremely untrustworthy	97 (47.1%)	
Untrustworthy	45 (21.8%)	
Neutral	42 (20.4%)	
Trustworthy	16 (7.8%)	
Extremely trustworthy	6 (2.9%)	
Trustworthiness: Books and magazines		Participants who rated (*n* = 216)
Extremely untrustworthy	27 (12.5%)	
Untrustworthy	38 (17.6%)	
Neutral	62 (28.7%)	
Trustworthy	71 (32.9%)	
Extremely trustworthy	18 (8.3%)	

Perceived effectiveness refers to participants’ self-reported rating of how effective they found their supplements; clinical efficacy was not assessed. Trustworthiness was rated on a slider recorded from 0 to 5; values of 0 and 1 were combined as ‘extremely untrustworthy’ (see Methods). Denominators for trustworthiness are the numbers of participants who rated each source (3 to 20 participants did not rate a given source).

The most common sources of information regarding menopause symptom management were healthcare providers (*n* = 156, 69.0%), and the internet, online articles, blogs and websites (*n* = 153, 67.7%). Social media influencers were the least trusted information sources, with 47.1% of the 206 respondents who rated this source (*n* = 97) ranking this source as “extremely untrustworthy”, while healthcare providers were the most trusted sources, with 75.3% of the 223 respondents who rated this source (*n* = 168) rating healthcare providers as “trustworthy” or “extremely trustworthy”.

The overall perceived effectiveness rating cross-tabulated by type of supplement used is presented in Supplementary Table S2. Because the questionnaire contained a single overall effectiveness item rather than a rating for each product, these figures describe the overall rating given by users of each product type and cannot be attributed to any specific product. Among products reported by at least 25 users, the proportion of users rating their supplements as somewhat or very effective ranged from 36.0% among calcium users (9 of 25 who answered) and 40.0% among vitamin D users (26 of 65) to 52.3% among magnesium users (34 of 65) and 57.1% among users of other vitamins (16 of 28); the corresponding figures were 46.7% for multivitamins (28 of 60), 50.9% for probiotics (27 of 53), and 46.2% for omega-3 fatty acids (12 of 26).

### Correlates of supplement type

Exploratory Spearman correlations between each supplement type and age, education, income, and smoking status are presented in Supplementary Table S3. Of the 80 correlations tested, 13 reached nominal significance (*p* < 0.05), all with small coefficients (rho 0.14 to 0.22). None remained significant after false discovery rate adjustment (all q > 0.05). The nominally significant correlations included positive associations between never or former smoking and use of multivitamins, vitamin D, magnesium, probiotics, calcium, and multiminerals; between age and calcium use; and between income and probiotic use. Given the number of tests, the small coefficients, and the small number of users of several products, these results should be regarded as descriptive.

### Multivariable logistic regression

In the multivariable logistic regression model (*n* = 224 complete cases, 138 users; Table 6), smoking status was independently associated with current supplement use for any purpose (Wald test for smoking, *p* = 0.012). Compared with current smokers, former smokers (aOR 2.98, 95% CI 1.21 to 7.34) and never smokers (aOR 3.92, 95% CI 1.59 to 9.65) had higher odds of supplement use. Participants aged 61–65 had higher odds of supplement use than those aged 45–50 (aOR 7.13, 95% CI 1.47 to 34.62); this estimate is based on 17 users and 2 non-users and its confidence interval is correspondingly wide. Education, income, and family history of cancer were not associated with supplement use. The overall model was statistically significant (likelihood ratio chi-square 22.34, 12 degrees of freedom, *p* = 0.034) but explained little of the variation in supplement use (McFadden pseudo-R^2^ 0.075). Variance inflation factors were all below 2.72 and the Hosmer-Lemeshow test indicated adequate calibration (chi-square 6.59, 8 degrees of freedom, *p* = 0.582). In the sensitivity analysis with menopause-targeted use (item 8) as the outcome, none of the covariates was associated with the outcome (former smokers aOR 1.21, 95% CI 0.44 to 3.33; never smokers aOR 1.66, 95% CI 0.62 to 4.49; Wald test for smoking *p* = 0.487; Supplementary Table S4).

**Table 6:** Multivariable logistic regression of current supplement use for any purpose (item 9) on participant characteristics (*n* = 224 complete cases; 138 users).

Variable	Adjusted OR	95% CI	*p*-value
Age group (years)			
45–50	1.00 (reference)		
51–55	1.22	0.63 to 2.36	0.555
56–60	0.73	0.32 to 1.67	0.453
61–65	7.13	1.47 to 34.62	0.015
Education			
High school diploma/GED or less	1.00 (reference)		
Some college/vocational training/Associate's degree	0.69	0.26 to 1.79	0.445
Bachelor's degree	1.00	0.34 to 2.97	0.998
Master's/Doctorate degree	0.58	0.18 to 1.87	0.364
Annual household income			
$0 to $49,999	1.00 (reference)		
$50,000 to $99,999	1.22	0.60 to 2.51	0.582
$100,000 to $199,999	1.38	0.62 to 3.07	0.424
$200,000 or more	1.18	0.38 to 3.64	0.771
Smoking status			0.012‡
Current smoker	1.00 (reference)		
Former smoker	2.98	1.21 to 7.34	0.018
Never smoked	3.92	1.59 to 9.65	0.003
Family history of cancer			
No	1.00 (reference)		
Yes	1.00	0.56 to 1.77	0.997

All covariates were entered simultaneously. OR, odds ratio; CI, confidence interval. Reference categories: 45–50 years; high school diploma/GED or less (the two participants with less than high school education were combined with this category); household income $0 to $49,999; current smoker; no family history of cancer. Race/ethnicity was not included because of sparse categories. ‡ Wald test for the joint effect of smoking status (2 degrees of freedom). Model likelihood ratio chi-square 22.34 (12 df), *p* = 0.034; McFadden pseudo-R^2^ 0.075; maximum variance inflation factor 2.72; Hosmer-Lemeshow chi-square 6.59 (8 df), *p* = 0.582. Two participants were excluded for missing income or family history. The estimate for the 61–65 year group is based on 17 users and 2 non-users.

## Discussion

This cross-sectional study examined dietary supplement use, perceptions of MHT, and information sources related to menopause symptom management among a self-selected online sample of midlife participants experiencing peri-menopausal or menopausal symptoms. Several key findings emerged. First, current use of at least one dietary supplement for any purpose was common (61.1% of respondents), whereas self-reported menopause-targeted supplement use was less common (28.9%); fewer than half of current supplement users reported using supplements to manage menopause symptoms. The most frequently reported supplements were multivitamins, vitamin D, magnesium, and probiotics, whereas menopause-specific botanical products such as black cohosh, evening primrose oil, red clover, and soy isoflavones were less commonly used. Additionally, awareness of MHT was relatively high, but participants reported substantial concerns about potential risks, particularly breast cancer, blood clotting, stroke, and heart disease. Healthcare providers and online information were the most reported sources of information, while social media influencers were less commonly used and were viewed as the least trustworthy source. Smoking status was the only demographic or health-related characteristic significantly associated with overall supplement use, with former smokers and never smokers more likely to report supplement use than current smokers; this association was not observed when supplement use was defined by the menopause-specific item.

The high prevalence of supplement use observed in this sample is consistent with previous research showing that many women seek complementary and alternative approaches for menopause symptom management (8, 12, 13, 15–17). Earlier studies from different regions have reported variable rates of complementary and alternative medicine use among menopausal women, ranging from approximately one-third to more than two-thirds of participants (12–17). The 61.1% rate observed in the present study is at the higher end of previously reported estimates, but two features of this study limit the comparison. First, participants were recruited through menopause support groups, health forums, and social media using voluntary response sampling; women with a particular interest in menopause, supplements, or wellness were therefore more likely to participate, and the prevalence estimate applies to this self-selected sample rather than to the general population of perimenopausal or postmenopausal women. Second, some prior studies assessed broad complementary and alternative medicine use, including herbal remedies, exercise, meditation, and prayer, whereas the present study focused specifically on dietary supplements, and the 61.1% estimate describes current supplement use for any purpose rather than use for menopause symptoms. The findings nevertheless indicate that supplements are a common component of self-care among symptomatic midlife women and should be routinely discussed in clinical encounters.

A central question raised by these results is whether the most frequently used supplements were being taken for menopausal symptoms or represent general supplement consumption. The two supplement items in the questionnaire allow a partial answer. Just under half of current supplement users (65 of 138) reported using supplements to manage menopause symptoms, and their product profile differed from that of the remaining users: they more often reported black cohosh, other herbal or botanical products, and probiotics, whereas users who did not report menopause-targeted use more often reported multivitamins and calcium, products typically taken for general health or bone health. This pattern suggests that a substantial share of the supplement use observed in this sample, and probably a substantial share of the multivitamin and vitamin D use in particular, reflects general health maintenance rather than symptom-targeted treatment. Even among those reporting menopause-targeted use, however, vitamins and minerals were more commonly reported than botanical products with a menopause-specific evidence base.

The types of supplements used in this sample therefore differed from patterns reported in some earlier studies. Previous research from North America, Europe, and Australia has often identified black cohosh, soy isoflavones, red clover, phytoestrogen-containing products, and evening primrose oil among the more frequently used products for symptoms such as hot flushes and night sweats (13, 14, 16, 18, 19). Several hypotheses could account for the difference, none of which can be tested with the present data: changes in consumer behavior over time, broader wellness trends and greater public attention to vitamin D and magnesium, differences in how participants interpreted the term 'supplement', and the inclusion in the present sample of general supplement users as described above. Because the survey relied on self-report and did not ask participants to indicate the intended purpose of each product, the present findings cannot establish whether each supplement was being used exclusively or primarily for menopause-related symptoms. Future studies should distinguish between general supplement use and symptom-targeted use at the level of the individual product and should collect information on dose, formulation, duration, and symptom indication.

Although many participants reported supplement use, perceived effectiveness was mixed. Among supplement users who answered the item, 47.0% rated their supplements as somewhat or very effective, 27.8% were neutral, and 25.2% rated them as ineffective. Perceived effectiveness was higher among users reporting menopause-targeted use (56.2%) than among those who did not (35.3%), which is consistent with the latter group not taking supplements for symptom relief in the first place. Among the most commonly used products, the overall ratings given by multivitamin and vitamin D users were lower than those given by magnesium and probiotic users (Supplementary Table S2), although the questionnaire's single overall rating cannot be attributed to specific products. These findings should be interpreted cautiously because perceived effectiveness is not equivalent to clinical efficacy; the study did not measure symptom severity, symptom change, or any validated menopause symptom scale, and perceived benefit may be influenced by expectations, concurrent lifestyle changes, placebo effects, symptom fluctuation, or simultaneous use of multiple products. Evidence for supplements in menopause symptom management remains heterogeneous. Meta-analyses have suggested potential benefit for some phytoestrogen-containing products and black cohosh in reducing vasomotor symptoms (20, 21), and individual trials have reported improvements with selected multi-ingredient nutraceutical formulations (22). However, reviews and clinical literature continue to emphasize that evidence is inconsistent for many natural products and that safety, product quality, interactions, and contraindications require consideration (2, 8, 23, 24). The current study adds to this literature by showing that the supplements most commonly used by respondents were not necessarily those with the most menopause-specific evidence base.

The findings on MHT awareness and perceptions provide additional context for supplement use. Most respondents were aware of MHT, and perceptions were most often neutral or positive. However, concerns about breast cancer, blood clotting, stroke, and heart disease were common. This pattern is consistent with the long-standing influence of the Women's Health Initiative (WHI) findings on public and clinical perceptions of menopausal hormone therapy (4, 5, 25). Initial WHI reports contributed to concern regarding MHT use, although subsequent analyses have emphasized the importance of age, time since menopause onset, baseline risk, treatment type, and individual contraindications when evaluating the benefit-risk profile of MHT (4, 6, 11, 26, 27). Current evidence supports individualized decision-making, particularly for symptomatic women younger than 60 years or within 10 years of menopause onset who do not have contraindications to hormone therapy (6, 11). The present study shows that concerns about MHT and supplement use coexist in this sample; it did not test, and cannot establish, whether concern about MHT leads women to use supplements. Whether these concerns contribute to interest in non-prescription alternatives is a hypothesis that would require a study designed to examine treatment decision-making directly.

The demographic differences observed in MHT awareness also warrant attention. MHT awareness was significantly associated with race/ethnicity, education, annual income, and smoking status. Participants identifying as Black/African American, those with lower educational attainment, those with lower annual income, and current smokers showed comparatively lower awareness; the observation in the single Native Hawaiian or other Pacific Islander participant cannot be interpreted. These findings are consistent with prior research suggesting that knowledge and attitudes toward MHT may vary according to sociodemographic factors, previous knowledge, healthcare experience, and cultural context (28–33). Because several racial and ethnic groups were represented by very few participants, these comparisons rest on small numbers and exact tests, and larger and more diverse studies are needed to determine whether these patterns are consistent and to identify cultural or healthcare-access factors that may shape awareness and decision-making about menopause treatment.

The association between smoking status and supplement use was the only association with a participant characteristic that was consistently observed in this study. Former smokers and never smokers had significantly higher odds of any supplement use than current smokers, while education, income, and family history of cancer were not significant predictors in the logistic regression model. Two features of this finding call for a cautious interpretation. First, the association was present only when supplement use was defined broadly; when the outcome was menopause-targeted use, smoking status was not associated with the outcome, which suggests that the association relates to general supplement use rather than to menopause symptom management specifically. Second, the model adjusted only for age, education, income, and family history of cancer. One hypothesis is that the association reflects broader health behavior patterns, whereby individuals who do not currently smoke may be more likely to engage in preventive health behaviors or wellness-oriented self-care practices. This hypothesis is plausible but was not tested. Smoking status may equally be associated with unmeasured factors, including access to healthcare, general health status, comorbidities, physical activity, medication use, symptom severity, health literacy, and other preventive behaviors, none of which were captured by the questionnaire. Future research should examine whether smoking status remains associated with supplement use after adjustment for these behavioral, psychosocial, and clinical variables.

Information sources represent another important finding. Healthcare providers and online articles, blogs, and websites were the most frequently reported sources of information about menopause symptom management. Healthcare providers were also rated as the most trustworthy source, while social media influencers were the least trusted. These findings are broadly consistent with prior studies showing that menopausal women may obtain information about complementary and alternative treatments from healthcare professionals, printed materials, online sources, and informal networks (17, 34). This has practical implications; although participants may seek information from online sources, they appear to place substantial trust in clinicians. Healthcare providers therefore have an important opportunity to ask proactively about supplement use, provide balanced counseling, discuss evidence and safety, and correct misinformation. This is especially important because patients may not spontaneously disclose supplement use, and some products may interact with medications or be inappropriate for individuals with specific medical histories. The coexistence of high internet use and high trust in clinicians also suggests that evidence-based digital resources may be useful adjuncts to clinical counseling.

### Limitations

This study has several limitations. First, the cross-sectional design prevents conclusions about causality or temporal relationships. The study can identify associations between participant characteristics and supplement use, but it cannot determine whether these characteristics influence supplement use or whether supplement use affects menopause symptoms. Second, participants were recruited online through menopause support groups, health forums, social media, and newsletters using voluntary response sampling. This is the most important limitation for the prevalence estimates: individuals with a strong interest in menopause, supplements, or women's health were more likely to participate, and the online format may have excluded individuals with limited digital access or lower digital literacy. The prevalence of supplement use reported here therefore describes this self-selected sample and should not be generalized to the broader population of perimenopausal or postmenopausal women. The sample was also predominantly White/Caucasian and highly educated, which further limits generalizability to more diverse populations. Third, women with a history of breast cancer, heart disease, or blood clots were excluded. Although this restricted the sample to women for whom MHT is a plausible option, these conditions are directly relevant to MHT decision-making and may themselves shape perceptions of MHT and interest in supplements; the findings therefore do not describe this clinically important group, and the proportion of women reporting MHT concerns may differ in a sample that includes them.

Fourth, the measurement of supplement use has limitations. The two supplement items measure different constructs: item 9 captures current use of specific products for any purpose, whereas item 8 captures self-reported use of supplements for menopause symptoms. The two estimates (61.1% and 28.9%) therefore describe different quantities and should not be read as bounds on a single quantity. Because item 9 did not ask the purpose for which each product was taken, products taken specifically for menopause symptoms cannot be identified at the product level, and the comparison of product profiles between users who did and did not report menopause-targeted use is indirect. Participants may also have interpreted the term 'supplement' differently. Fifth, the questionnaire was developed for this study and was not formally validated or pilot tested. It did not measure symptom type, severity, or impact using a validated instrument, so it is not possible to relate supplement use, MHT use, or perceived benefit to symptom burden; it also did not distinguish perimenopausal from postmenopausal participants, although symptom patterns, treatment use, and supplement behaviors may differ between these phases. The perceived effectiveness item was a single overall rating, which cannot be attributed to individual products. Sixth, the analyses of associations were exploratory. Several comparisons involved small cell counts, for which exact tests were used, and the correlation analyses across 20 supplement types involved 80 tests; none of the nominally significant correlations survived adjustment for multiple testing, and the logistic regression estimate for the oldest age group rests on very few non-users. No *a priori* sample size calculation was performed, and the study was not powered to detect associations of a prespecified size. Seventh, self-reported data are subject to recall and reporting bias. Finally, the present study did not collect detailed information on product dose, formulation, brand, adherence, concurrent supplement use, medication use, or symptom severity. These factors are important because supplement composition and quality can vary substantially, and menopause symptoms may fluctuate over time.

Despite these limitations, this study contributes to the growing literature on menopause symptom management by describing contemporary supplement use, MHT perceptions, and information-seeking patterns among a sample of symptomatic midlife participants. The findings indicate that dietary supplements were widely used in this sample, that fewer than half of users attributed their use to menopause symptoms, that concerns about MHT persisted despite relatively high awareness, and that clinicians remained central trusted sources of information. These results underscore the need for patient-centered counseling that addresses both evidence-based MHT decision-making and the safe, informed use of supplements. Future research should use larger, more diverse, and population-based samples to better characterize the supplements used across racial, ethnic, socioeconomic, and geographic groups. Longitudinal studies are needed to examine whether supplement use changes over the menopause transition and whether perceived benefits correspond to changes in validated symptom scores. Future studies should also ask about the intended purpose of each product and assess dose, formulation, duration, safety concerns, medication interactions, and communication with healthcare providers. Such research would help clarify how women make treatment decisions and how clinicians can better support informed, individualized menopause care.

## Conclusion

In this self-selected online sample of women experiencing peri-menopausal or menopausal symptoms, current dietary supplement use for any purpose was common (61.1%), and 28.9% of participants reported using supplements specifically to manage menopause symptoms. Multivitamins, vitamin D, magnesium, and probiotics were reported most frequently, and menopause-specific botanical products were concentrated among participants reporting menopause-targeted use. MHT awareness was high, but concerns about potential risks remained common; the study did not examine whether these concerns influence supplement use. Healthcare providers and online sources were the leading sources of information, while social media influencers were viewed as comparatively untrustworthy. Smoking status was associated with any supplement use but not with menopause-targeted use, and this association should be interpreted as hypothesis-generating. These findings should not be generalized beyond the sample studied. They nevertheless highlight the importance of routine clinical discussion about supplement use, evidence-based counseling on menopause treatment options, and further research using probability-based samples and validated symptom measures to characterize the safety, effectiveness, and decision-making contexts of supplement use during menopause.

## Data Availability

The raw data supporting the conclusions of this article will be made available by the authors, without undue reservation.
