# Supplementary material for: Dietary supplements are commonly used for menopause symptoms amid concerns about hormone therapy: a cross-sectional study
Source: Front Glob Womens Health. 2026 Oct 13;7:1924461. doi: 10.3389/fgwh.2026.1924461 (PMC13714707; doi:10.3389/fgwh.2026.1924461)
Supplement: Supplementary file 1 [file Table1.docx]

**Supplementary Material**

*Dietary Supplements Are Commonly Used for Menopause Symptoms Amid Concerns About Hormone Therapy: A Cross-Sectional Study*

Susanne Mitschke. Frontiers in Global Women’s Health, manuscript 1924461.

**Contents**

•	Supplementary Table S1. Participant flow and item-level missing data

•	Supplementary Table S2. Overall perceived effectiveness rating by type of supplement used

•	Supplementary Table S3. Spearman correlations between supplement type and participant characteristics, with false discovery rate adjustment

•	Supplementary Table S4. Sensitivity analyses: alternative definitions of supplement use and exclusion of participants with a blank eligibility item

•	Supplementary Table S5. Study questionnaire

**Supplementary Table S1.** Participant flow and item-level missing data.

**A. Participant flow**

| **Step** | **n** |
| --- | --- |
| Responses recorded in Qualtrics, July 21 to August 12, 2024 | 230 |
| Excluded: test response generated through the survey preview function before recruitment | 1 |
| Responses from participants | 229 |
| Excluded: age category 40-44 selected (outside eligibility criterion of 45-65 years) | 3 |
| Analytic sample | 226 |
| Answered “yes” to menopausal status eligibility item | 215 |
| Left eligibility item blank (retained in primary analysis; excluded in sensitivity analysis) | 11 |
| Did not tick consent checkbox but proceeded through the questionnaire (retained) | 1 |
| Sensitivity sample (eligibility item answered “yes”) | 215 |

All 230 recorded responses had reached the end of the questionnaire (100% progress). Because responses were anonymous and IP addresses were not recorded, duplicate responses could not be checked.

**B. Item-level missing data in the analytic sample (n=226)**

| **Item** | **Answered** | **Missing n (%)** |
| --- | --- | --- |
| Consent item | 225 | 1 (0.4%) |
| Menopause status (Q1) | 215 | 11 (4.9%) |
| Age | 226 | 0 (0.0%) |
| Race or ethnicity | 226 | 0 (0.0%) |
| Education | 226 | 0 (0.0%) |
| Annual household income | 225 | 1 (0.4%) |
| Smoking status | 226 | 0 (0.0%) |
| Family history of cancer | 225 | 1 (0.4%) |
| Supplement use for menopause symptoms (Q8) | 225 | 1 (0.4%) |
| Supplement types (Q9) | 212 | 14 (6.2%) |
| Duration of use (Q10) | 223 | 3 (1.3%) |
| Frequency of use (Q11) | 223 | 3 (1.3%) |
| MHT awareness | 226 | 0 (0.0%) |
| MHT use | 226 | 0 (0.0%) |
| Perception of MHT | 226 | 0 (0.0%) |
| Concerns about MHT | 225 | 1 (0.4%) |
| Information sources | 226 | 0 (0.0%) |
| Trust: healthcare providers | 223 | 3 (1.3%) |
| Trust: family and friends | 218 | 8 (3.5%) |
| Trust: internet | 221 | 5 (2.2%) |
| Trust: social media | 206 | 20 (8.8%) |
| Trust: books and magazines | 216 | 10 (4.4%) |
| Factors influencing choice (Q18) | 224 | 2 (0.9%) |
| Perceived effectiveness (Q19) | 224 | 2 (0.9%) |

“Missing” means the item was left blank. Items with a response option “I don’t use supplements” (items 9, 10, 11, 18, 19) are counted as answered when that option was selected. All analyses used complete cases for the variables involved; the denominator is stated for every estimate in the main text and tables.

**Supplementary Table S2.** Overall perceived effectiveness rating cross-tabulated by type of supplement used (supplement users, n=138).

| **Supplement type** | **Users** | **Very ineffective n (%)** | **Somewhat ineffective n (%)** | **Neutral n (%)** | **Somewhat effective n (%)** | **Very effective n (%)** | **Did not answer** |
| --- | --- | --- | --- | --- | --- | --- | --- |
| Multivitamin | 77 | 8 (13.3%) | 9 (15.0%) | 15 (25.0%) | 25 (41.7%) | 3 (5.0%) | 17 |
| Vitamin D | 73 | 13 (20.0%) | 9 (13.8%) | 17 (26.2%) | 21 (32.3%) | 5 (7.7%) | 8 |
| Magnesium | 71 | 8 (12.3%) | 7 (10.8%) | 16 (24.6%) | 31 (47.7%) | 3 (4.6%) | 6 |
| Probiotics | 57 | 6 (11.3%) | 5 (9.4%) | 15 (28.3%) | 24 (45.3%) | 3 (5.7%) | 4 |
| Other vitamins | 31 | 4 (14.3%) | 4 (14.3%) | 4 (14.3%) | 13 (46.4%) | 3 (10.7%) | 3 |
| Calcium | 27 | 3 (12.0%) | 4 (16.0%) | 9 (36.0%) | 6 (24.0%) | 3 (12.0%) | 2 |
| Omega-3 fatty acids | 27 | 5 (19.2%) | 2 (7.7%) | 7 (26.9%) | 10 (38.5%) | 2 (7.7%) | 1 |
| Black cohosh | 14 | 0 (0.0%) | 2 (14.3%) | 1 (7.1%) | 9 (64.3%) | 2 (14.3%) | 0 |
| Other dietary supplement | 14 | 2 (14.3%) | 1 (7.1%) | 3 (21.4%) | 5 (35.7%) | 3 (21.4%) | 0 |
| Other herbal supplement | 13 | 0 (0.0%) | 0 (0.0%) | 0 (0.0%) | 11 (84.6%) | 2 (15.4%) | 0 |
| Multimineral | 11 | 0 (0.0%) | 3 (30.0%) | 1 (10.0%) | 6 (60.0%) | 0 (0.0%) | 1 |
| Flaxseed | 9 | 0 (0.0%) | 0 (0.0%) | 3 (33.3%) | 5 (55.6%) | 1 (11.1%) | 0 |
| Other minerals | 7 | 0 (0.0%) | 0 (0.0%) | 0 (0.0%) | 4 (66.7%) | 2 (33.3%) | 1 |
| Evening primrose oil | 5 | 0 (0.0%) | 0 (0.0%) | 0 (0.0%) | 3 (75.0%) | 1 (25.0%) | 1 |
| Traditional Chinese Medicine herbs | 4 | 0 (0.0%) | 1 (25.0%) | 1 (25.0%) | 1 (25.0%) | 1 (25.0%) | 0 |
| Red clover | 4 | 0 (0.0%) | 0 (0.0%) | 0 (0.0%) | 1 (33.3%) | 2 (66.7%) | 1 |
| Valerian root | 4 | 1 (25.0%) | 0 (0.0%) | 1 (25.0%) | 1 (25.0%) | 1 (25.0%) | 0 |
| Ginseng | 3 | 0 (0.0%) | 1 (33.3%) | 0 (0.0%) | 1 (33.3%) | 1 (33.3%) | 0 |
| Soy isoflavones | 2 | 0 (0.0%) | 0 (0.0%) | 0 (0.0%) | 2 (100.0%) | 0 (0.0%) | 0 |
| St. John’s Wort | 2 | 0 (0.0%) | 0 (0.0%) | 0 (0.0%) | 2 (100.0%) | 0 (0.0%) | 0 |

The questionnaire contained a single overall item (“how effective do you find the dietary supplements in managing your menopause symptoms?”) rather than a rating for each product. Each row therefore shows the overall rating given by users of that product type; because most users reported several products (median 3), the ratings cannot be attributed to any specific product. Percentages use as the denominator the number of users of each product who gave a substantive answer to the effectiveness item (users minus “did not answer”, which includes those who selected “I don’t use supplements”). Products used by fewer than 10 participants are shown for completeness only. This table replaces Table 7 of the original submission; the original percentages used all users of each product as the denominator, including those who did not answer.

**Supplementary Table S3.** Spearman rank correlations between use of each supplement type and participant characteristics (n=226), with Benjamini-Hochberg false discovery rate adjustment across all 80 tests.

| **Supplement type** | **Users** | **Age rho (p; q)** | **Education rho (p; q)** | **Income rho (p; q)** | **Smoking rho (p; q)** |
| --- | --- | --- | --- | --- | --- |
| Multivitamin | 77 | 0.06 (0.395; 0.847) | 0.08 (0.216; 0.752) | 0.10 (0.124; 0.551) | 0.15 (0.021; 0.190) |
| Vitamin D | 73 | 0.05 (0.496; 0.862) | 0.05 (0.433; 0.847) | 0.06 (0.352; 0.808) | 0.20 (0.003; 0.074) |
| Magnesium | 71 | 0.00 (0.989; 0.998) | 0.09 (0.158; 0.664) | 0.07 (0.305; 0.808) | 0.14 (0.042; 0.261) |
| Probiotics | 57 | -0.00 (0.998; 0.998) | 0.09 (0.203; 0.752) | 0.19 (0.004; 0.081) | 0.17 (0.013; 0.184) |
| Other vitamins | 31 | -0.05 (0.455; 0.847) | 0.03 (0.630; 0.934) | 0.03 (0.615; 0.934) | 0.03 (0.705; 0.934) |
| Calcium | 27 | 0.22 (<0.001; 0.072) | 0.04 (0.558; 0.911) | -0.02 (0.821; 0.934) | 0.15 (0.023; 0.190) |
| Omega-3 fatty acids | 27 | 0.11 (0.115; 0.541) | 0.00 (0.962; 0.986) | -0.03 (0.685; 0.934) | 0.04 (0.544; 0.906) |
| Black cohosh | 14 | -0.02 (0.819; 0.934) | -0.03 (0.695; 0.934) | -0.01 (0.872; 0.935) | -0.06 (0.406; 0.847) |
| Other dietary supplement | 14 | 0.02 (0.764; 0.934) | 0.03 (0.627; 0.934) | 0.01 (0.834; 0.934) | 0.07 (0.305; 0.808) |
| Other herbal supplement | 13 | -0.08 (0.210; 0.752) | 0.09 (0.176; 0.703) | 0.02 (0.733; 0.934) | 0.07 (0.329; 0.808) |
| Multimineral | 11 | 0.11 (0.085; 0.423) | 0.05 (0.426; 0.847) | 0.02 (0.791; 0.934) | 0.15 (0.024; 0.190) |
| Flaxseed | 9 | 0.20 (0.002; 0.074) | 0.08 (0.234; 0.779) | -0.01 (0.877; 0.935) | 0.12 (0.067; 0.356) |
| Other minerals | 7 | -0.05 (0.490; 0.862) | -0.01 (0.853; 0.934) | -0.03 (0.614; 0.934) | 0.02 (0.729; 0.934) |
| Evening primrose oil | 5 | -0.05 (0.487; 0.862) | -0.06 (0.405; 0.847) | -0.02 (0.769; 0.934) | -0.05 (0.454; 0.847) |
| Traditional Chinese Medicine herbs | 4 | 0.03 (0.643; 0.934) | 0.04 (0.533; 0.906) | 0.02 (0.768; 0.934) | 0.13 (0.053; 0.305) |
| Red clover | 4 | 0.08 (0.257; 0.784) | -0.01 (0.903; 0.950) | -0.02 (0.784; 0.934) | 0.02 (0.761; 0.934) |
| Valerian root | 4 | 0.08 (0.257; 0.784) | 0.15 (0.029; 0.210) | 0.02 (0.768; 0.934) | 0.07 (0.265; 0.784) |
| Ginseng | 3 | 0.02 (0.744; 0.934) | 0.01 (0.847; 0.934) | -0.01 (0.827; 0.934) | -0.05 (0.448; 0.847) |
| Soy isoflavones | 2 | 0.16 (0.016; 0.184) | -0.01 (0.931; 0.968) | -0.06 (0.342; 0.808) | -0.06 (0.354; 0.808) |
| St. John’s Wort | 2 | 0.16 (0.016; 0.184) | 0.14 (0.035; 0.233) | 0.07 (0.277; 0.792) | -0.06 (0.354; 0.808) |

rho, Spearman correlation coefficient between use of the supplement (0/1) and the ordinal characteristic. Age: 45-50 < 51-55 < 56-60 < 61-65. Education: high school diploma/GED or less < some college/vocational training/associate’s degree < bachelor’s degree < master’s/doctorate degree. Income: four ordered categories. Smoking: current smoker < former smoker < never smoked, so a positive coefficient indicates higher use among former and never smokers. p, unadjusted p-value; q, Benjamini-Hochberg false discovery rate adjusted q-value across all 80 tests. Of 80 tests, 13 had unadjusted p<0.05 and 0 had q<0.05. Inferential interpretation is restricted to supplement types with at least 10 users; results for rarer products are shown for completeness only. This table replaces Table 8 of the original submission.

**Supplementary Table S4.** Sensitivity analyses.

**A. Prevalence of supplement use under alternative definitions and samples**

| **Definition and sample** | **n / N** | **%** | **95% CI** |
| --- | --- | --- | --- |
| Primary: at least one supplement type selected in item 9 (analytic sample) | 138 / 226 | 61.1 | 54.6 to 67.2 |
| Direct item 8 answered “yes” (analytic sample; 1 non-response to item 8) | 65 / 225 | 28.9 | 23.4 to 35.1 |
| Item 8 “yes” and at least one supplement type selected (analytic sample) | 65 / 226 | 28.8 | 23.3 to 35.0 |
| Primary definition, eligibility item answered “yes” (sensitivity sample) | 131 / 215 | 60.9 | 54.3 to 67.2 |
| Direct item 8 “yes”, eligibility item answered “yes” (sensitivity sample) | 63 / 214 | 29.4 | 23.7 to 35.9 |

Concordance of the two items in the analytic sample: item 8 “yes” and at least one product selected, 65; item 8 “yes” and no product, 0; item 8 “no” and at least one product, 72; item 8 “no” and no product, 88; item 8 blank and at least one product, 1. 14 participants left item 9 blank (all answered “no” to item 8) and were classified as non-users. 95% CI, Wilson interval.

**B. Participant characteristics by menopause-targeted supplement use (item 8 “yes” versus “no”; n=225)**

| **Characteristic** | **p-value** |
| --- | --- |
| Age group (years) | 0.804 |
| Race or ethnicity | 0.292 |
| Education | 0.703 |
| Annual household income | 0.197 |
| Smoking status | 0.388 |
| Family history of cancer | 0.665 |
| *Smoking status, item 8 “yes” by category* | Current 7 (20.6%); former 22 (27.5%); never 36 (32.4%) |

p-values from Pearson’s chi-square test or Fisher’s exact test (Fisher-Freeman-Halton extension) where any expected cell count was below 5, as in Table 1 of the main text.

**C. Supplement types reported by users who affirmed menopause-targeted use (item 8 “yes”; n=65) and users who did not (item 8 “no”; n=73)**

| **Supplement type** | **Affirmed menopause-targeted use n (%)** | **Did not affirm n (%)** | **p-value** |
| --- | --- | --- | --- |
| Multivitamin | 26 (40.0%) | 51 (69.9%) | <0.001 |
| Vitamin D | 30 (46.2%) | 43 (58.9%) | 0.134 |
| Magnesium | 37 (56.9%) | 34 (46.6%) | 0.225 |
| Probiotics | 36 (55.4%) | 21 (28.8%) | 0.002 |
| Other vitamins | 10 (15.4%) | 21 (28.8%) | 0.060 |
| Calcium | 8 (12.3%) | 19 (26.0%) | 0.043 |
| Omega-3 fatty acids | 16 (24.6%) | 11 (15.1%) | 0.158 |
| Black cohosh | 12 (18.5%) | 2 (2.7%) | 0.002 |
| Any herbal or botanical product | 26 (40.0%) | 10 (13.7%) | <0.001 |
| Perceived effectiveness: somewhat or very effective | 36 (56.2%) of 64 who answered | 18 (35.3%) of 51 who answered | 0.015§ |

The one user who did not answer item 8 is excluded. Shown are the eight most frequently reported supplement types and a combined indicator for any herbal or botanical product (black cohosh, soy isoflavones, red clover, evening primrose oil, flaxseed, ginseng, St. John’s Wort, valerian root, traditional Chinese medicine herbs, other herbal supplement). p-values from Pearson’s chi-square test or Fisher’s exact test where any expected count was below 5; § Mann-Whitney U test on the five-level effectiveness rating. These comparisons are unadjusted for multiple testing.

**D. Multivariable logistic regression with menopause-targeted supplement use (item 8 “yes”) as the outcome**

| **Variable** | **Adjusted OR** | **95% CI** | **p-value** |
| --- | --- | --- | --- |
| **Age group (years)** |  |  |  |
| 45-50 | 1.00 (reference) |  |  |
| 51-55 | 0.90 | 0.45 to 1.80 | 0.767 |
| 56-60 | 0.70 | 0.27 to 1.78 | 0.450 |
| 61-65 | 0.93 | 0.29 to 3.00 | 0.903 |
| **Education** |  |  |  |
| High school diploma/GED or less | 1.00 (reference) |  |  |
| Some college/vocational training/Associate's degree | 1.15 | 0.40 to 3.29 | 0.790 |
| Bachelor's degree | 1.42 | 0.46 to 4.44 | 0.544 |
| Master's/Doctorate degree | 0.76 | 0.22 to 2.67 | 0.672 |
| **Annual household income** |  |  |  |
| $0 to $49,999 | 1.00 (reference) |  |  |
| $50,000 to $99,999 | 1.13 | 0.51 to 2.48 | 0.764 |
| $100,000 to $199,999 | 1.78 | 0.78 to 4.06 | 0.171 |
| $200,000 or more | 2.43 | 0.80 to 7.36 | 0.115 |
| **Smoking status** |  |  | 0.487‡ |
| Current smoker | 1.00 (reference) |  |  |
| Former smoker | 1.21 | 0.44 to 3.33 | 0.713 |
| Never smoked | 1.66 | 0.62 to 4.49 | 0.316 |
| **Family history of cancer** |  |  |  |
| No | 1.00 (reference) |  |  |
| Yes | 1.24 | 0.68 to 2.27 | 0.475 |

n=223 complete cases, 65 affirming menopause-targeted use. Same specification as Table 6 of the main text. ‡ Wald test for the joint effect of smoking status. Model likelihood ratio chi-square 8.72 (12 df), p=0.727; McFadden pseudo-R² 0.032; maximum variance inflation factor 2.72; Hosmer-Lemeshow chi-square 10.47 (8 df), p=0.234. With only 65 events for 12 model parameters, this model is underpowered and is presented for transparency only. In the primary model (Table 6) restricted to the sensitivity sample (n=214), the smoking estimates were essentially unchanged: former smoker aOR 3.03 (95% CI 1.22 to 7.52), never smoked aOR 3.87 (95% CI 1.57 to 9.55).

**Supplementary Table S5.** Study questionnaire, as administered in Qualtrics (July 21 to August 12, 2024).

**Section 1: Demographic Information**

1. Menopause status. Are you currently menopausal or peri-menopausal with symptoms? (Such as hot flashes, sleep disturbances, mood changes, vaginal dryness, changes in libido, weight changes, issues with cognitive functioning.)

*Response options: Yes; No*

2. Age. What is your age?

*Response options: 40-44; 45-50; 51-55; 56-60; 61-65; Other*

3. Race. What is your race or ethnicity?

*Response options: American Indian; Asian; Black/African American; Hispanic/Latino; Native American or Alaska Native; Native Hawaiian/other Pacific Islander; White/Caucasian; Other*

4. Education. What is the highest level of education you have completed?

*Response options: No degree; High school diploma or GED; Some college/vocational training/Associate’s degree; Bachelor’s degree; Master’s/Doctorate degree*

5. Income. What is your annual household income?

*Response options: $0 to $49,999; $50,000 to $99,999; $100,000 to $199,999; $200,000 or more*

6. Smoking status. Do you currently smoke, or have you ever smoked?

*Response options: Never smoked; Former smoker; Current smoker*

7. Family history of cancer. Do you have a family history of cancer?

*Response options: Yes; No*

**Section 2: Prevalence of Dietary Supplement Use**

8. Dietary supplement use. Are you currently using any dietary supplements to manage menopause symptoms?

*Response options: Yes; No*

9. Dietary supplement use. If you use supplements, please list the dietary supplements you are using: (select all that apply)

*Response options: Multivitamin; Multimineral; Traditional Chinese Medicine herbs; Black cohosh; Soy isoflavones; Red clover; Evening primrose oil; Vitamin D; Calcium; Magnesium; Omega-3 fatty acids; Probiotics; Flaxseed; Ginseng; St. John’s Wort; Valerian root; Other herbal supplement; Other vitamins; Other minerals; Other dietary supplement; I don’t use supplements*

10. Duration of use. If you use supplements, how long have you been using dietary supplements for menopause symptoms?

*Response options: Less than 1 month; 1-3 months; 4-6 months; 7-12 months; More than 1 year; I don’t use supplements*

11. Frequency of use. If you use supplements, how often do you take these dietary supplements?

*Response options: Daily; Weekly; Monthly; Occasionally; Only when I have symptoms; I don’t use supplements*

**Section 3: Perceptions and Misconceptions of Hormone Replacement Therapy (HRT)**

12. Awareness of HRT. Are you aware of Hormone Replacement Therapy (HRT) as a treatment option for menopause symptoms?

*Response options: Yes; No*

13. Usage of HRT. Are you currently using Hormone Replacement Therapy to manage menopause symptoms?

*Response options: Yes; No*

14. Perceptions of HRT. What is your overall perception of HRT?

*Response options: Very positive; Somewhat positive; Neutral; Somewhat negative; Very negative*

15. Concerns about HRT. Do you have any concerns about HRT? (Select all that apply)

*Response options: No concerns; Increased risk of breast cancer; Increased risk of heart disease; Increased risk of stroke; Increased risk of blood clots; Other*

**Section 4: Sources of Information About Dietary Supplements**

16. Primary sources of information. What are your primary sources of information regarding menopause symptom management? (Select all that apply)

*Response options: Healthcare providers; Family and friends; Internet // Online articles, blogs, and websites; Social media/influencers; Books and magazines; Other*

17. Trustworthiness of sources. How trustworthy do you consider the following sources of information in regards to menopause symptom management? (Rate each from 1 to 5, where 1 = Not trustworthy and 5 = Very trustworthy)

*Response options: Healthcare providers; Family and friends; Internet // Online articles, blogs, and websites; Social media/influencers; Books and magazines*

**Section 5: Factors Influencing Supplement Use**

18. Reasons for choosing supplements. If you use supplements, what factors influenced your choice of dietary supplements for menopause symptom management? (Select all that apply)

*Response options: Perceived efficacy; Safety; Recommendations from healthcare providers; Recommendations from family and friends; Recommendations from social media influencers; Marketing and advertisements; Personal research; Other; I don’t use supplements*

19. Effectiveness of supplements. If you use supplements, how effective do you find the dietary supplements in managing your menopause symptoms?

*Response options: Very effective; Somewhat effective; Neutral; Somewhat ineffective; Very ineffective; I don’t use supplements*

The questionnaire was preceded by the study information sheet and a consent item (“I consent to participate in this study”). The questionnaire uses the term hormone replacement therapy (HRT); the manuscript uses the contemporary term menopausal hormone therapy (MHT) to refer to the same treatment. Item 17 was implemented as a slider; the recorded values ranged from 0 to 5 (see Methods). In the recorded data, item 18 contained a single selection per participant.
